# Expression Level Dominance and Homeolog Expression Bias Upon Cold Stress in the F1 Hybrid Between the Invasive *Sphagneticola trilobata* and the Native *S. calendulacea* in South China, and Implications for Its Invasiveness

**DOI:** 10.3389/fgene.2022.833406

**Published:** 2022-05-19

**Authors:** Wei Wu, Wei Guo, Guangyan Ni, Longyuan Wang, Hui Zhang, Wei Lun Ng

**Affiliations:** ^1^ College of Horticulture and Landscape Architecture, Zhongkai University of Agriculture and Engineering, Guangzhou, China; ^2^ Key Laboratory of Vegetation Restoration and Management of Degraded Ecosystems, South China Botanical Garden, Chinese Academy of Sciences, Guangzhou, China; ^3^ China-ASEAN College of Marine Sciences, Xiamen University Malaysia, Sepang, Malaysia

**Keywords:** invasive species, hybrid weed, homeolog expression bias, transcriptomic shock, cold adaptation

## Abstract

The role of hybridization is significant in biological invasion, and thermotolerance is a trait critical to range expansions. The South American *Sphagneticola trilobata* is now widespread in South China, threatening the native *S. calendulacea* by competition and hybridization. Furthermore, upon formation, their F1 hybrid can quickly replace both parents. In this study, the three taxa were used as a model to investigate the consequences of hybridization on cold tolerance, particularly the effect of subgenome dominance in the hybrid. Upon chilling treatments, physiological responses and transcriptome profiles were compared across different temperature points to understand their differential responses to cold. While both parents showed divergent responses, the hybrid’s responses showed an overall resemblance to *S. calendulacea,* but the contribution of homeolog expression bias to cold stress was not readily evident in the F1 hybrid possibly due to inherent bias that comes with the sampling location. Our findings provided insights into the role of gene expression in differential cold tolerance, and further contribute to predicting the invasive potential of other hybrids between *S. trilobata* and its congeners around the world.

## Introduction

Natural hybridization is prevalent in plants and is regarded as an important driving force in a variety of evolutionary processes ranging from local adaptation to speciation (Stebbins, 1959; Rieseberg, 1995; Soltis and Soltis, 2009). With ever-increasing anthropogenic activities and human-induced ecological alterations, once geographically isolated species are increasingly brought together resulting in frequent interspecific hybridization events (McCann et al., 2018). In this regard, invasive alien species can rapidly recover from low genetic variation caused by the founder effect, or acquire adaptive alleles from resident species, by means of admixture and introgression. In fact, natural hybridization has been shown to be capable of facilitating rapid evolutionary changes at a contemporary timescale, and mounting evidences have lent support to its role as a promoter of invasiveness (Abbott, 1992; Petit et al., 2004; Ainouche et al., 2008; Schierenbeck and Ellstrand, 2008; Blair and Hufbauer, 2010). While heterosis in first-generation (F1) hybrids is generally considered to be transient due to reduced fertility, the ability for apomixis and clonal spread in many plant hybrids contribute significantly to their possible invasiveness. Some notoriously invasive plants known to be F1 hybrids include the cattail *Typha × glauca* in North America (a hybrid between the native *T. latifolia* and European invader *T. angustifolia*) (Ciotir et al., 2013) and the cordgrass *Spartina* × *townsendii* in northern Europe (a hybrid between the native small cordgrass, *S. maritima*, and the introduced smooth cordgrass, *S. alterniflora*) (Ainouche et al., 2004). When colonizing new habitats, these hybrids will usually be challenged by a variety of biotic or abiotic stresses including predators, cold/heat, drought, salinity, etc., but successful invaders would rapidly acclimatize to the environments (McCann et al., 2018).

The merger of divergent genomes within a hybrid can lead to immediate genome structure (e.g. inversion, translocation, and chromosomal fusion) and epigenetic alterations, causing extensive changes in gene expression (i.e. transcriptome shock), leading to phenotypic variations that are important for species establishment and diversification (Adams and Wendel, 2005; Hegarty et al., 2006; Doyle et al., 2008). Transcriptome shock invokes the disruption of parental expression patterns in the hybrids, which can have many manifestations (Wu et al., 2016). One major manifestation is expression level dominance (= non-additive gene expression), in which the expression level of the hybrid is not equivalent to the average of parental expression (Wendel, 2000; Jackson and Chen, 2010). Expression level dominance can be further categorized into transgressive expression (i.e. the total expression of the hybrid exceeds that of the parental expression range) and parental expression level dominance (i.e. the total expression of the hybrid is equal to one of the parents but significantly different from the other) (Wendel, 2000; Jackson and Chen, 2010). It is based on comparison between the total expression level of all homeologs in the hybrid and its corresponding parental expression levels (Wendel, 2000; Jackson and Chen, 2010). The other manifestation for transcriptome shock is homeolog expression bias (a.k.a. transcriptome dominance on a genome-wide basis), which concerns the relative expression of the diverged parental homeologs in the hybrid, irrespective of the overall expression level for a given homologous locus (Wendel, 2000; Jackson and Chen, 2010). Transcriptome shock characterized by expression level dominance or homeolog expression bias have been reported in many allopolyploids or hybrids, including Arabidopsis (Wang et al., 2006), rice (Wu et al., 2016), cotton (Zheng et al., 2016), wheat (Mutti et al., 2017), *Mimulus* (Edger et al., 2018), *Tragopogon* (Buggs et al., 2011), and *Spartina* (Chelaifa et al., 2010). Some changes in gene expression have been thought to be responsible for phenotypic variation between newly formed allopolyploids and their parental species, and may be the primary source of phenotypic novelty (Bird et al., 2018).

*Sphagneticola trilobata* (L.) Pruski (synonym: *Wedelia trilobata*; 2n = 4x = 56) is a creeping perennial herb native to South America. Being one of the IUCN’s 100 worst invasive species (Lowe and Boudjelas, 2000), *S. trilobata* is now widespread in many tropical and subtropical areas around the world. Since its introduction as groundcover in the 1970s, the species has escaped from gardens and spread rampantly in Southern China. Owing to its vigorous vegetative reproduction, this fast-growing herb has become a notorious weed, crowding out and preventing the regeneration of native plants by competing for nutrients, light, and water (https://www.cabi.org/isc/datasheet/56714). Threatened by this exotic weed, the abundance and distribution of the congeneric native species *S. calendulacea* (L.) Pruski (synonym: *Wedelia chinensis* Merr; 2n = 50) have been decreasing rapidly over the past decades. Unlike the invasive species, *S. calendulacea* has greater cold tolerance with a more northward distribution, but less drought tolerance with restricted occurrences in riverbanks, coastlands, and other moist habitats (Chen and Nicholas Hind, 2011). Recently, natural hybridization between *S. calendulacea* and *S. trilobata* has been recorded in the field, with subsequent molecular (Wu et al., 2013) and karyotype (Li et al., 2015) analyses showing that the hybrids were confined to the F1 generation. In South China, the F1 hybrid *via* rampant propagation would replace both the parental species rapidly once hybridization occurs (Wei Wu, pers. obs.). Considering the difference in cold tolerance between the two parental species*,* it is imperative to evaluate the performance of the hybrid under cold conditions and predict its consequences on future invasiveness. After all, cold tolerance has a direct effect on the geographic distributions of plant species (Pearce, 2001), including the range expansion of alien invasive species (Kelley, 2014). While extensive efforts have been undertaken to identify the molecular components of cold stress in model plants (Ding et al., 2019), the knowledge was not always transferable to other wild species (Zhang et al., 2004) and we are still far away from a comprehensive understanding of cold acclimation (Knight and Knight, 2012).

While extensive genetic studies associated with yield- or growth-related traits have been conducted for crop hybrids (e.g. in rice (Yu et al., 2020), wheat (Nadolska-Orczyk et al., 2017), cotton (Shahzad et al., 2019), peanut (Janila et al., 2013), and coffee (Marie et al., 2020)), studies on other traits related to ecological adaptations are lacking. Such a trend may be attributed to ambiguous parent ascertainment or the lack of genome assemblies associated with the hybrid systems (Edger et al., 2018). With increasing accessibility to next generation sequencing technologies, investigating ecological traits of non-model hybrid systems becomes more feasible (Buggs et al., 2012). In this study, we wanted to know how hybridization would affect cold responses in the F1 hybrids between the diploid *S. calendulacea* and the allotetraploid *S. trilobata* (Wu et al., 2010). To predict the invasion potential of the emerging hybrid, we simulated chilling stress on both parental species and the hybrid, and then characterized their physiological responses and transcriptome profiles across a temperature gradient. Using a time series RNA-seq analysis, we aimed to 1) unravel overall expression differentiation between the two *Sphagneticola* species and determine the extent of transcriptome shock in the F1 hybrid; and 2) determine the contribution of subgenome dominance to the overall cold tolerance in the F1 hybrid.

## Results

### Physiological Response to Cold Stress

**FIGURE 1:**
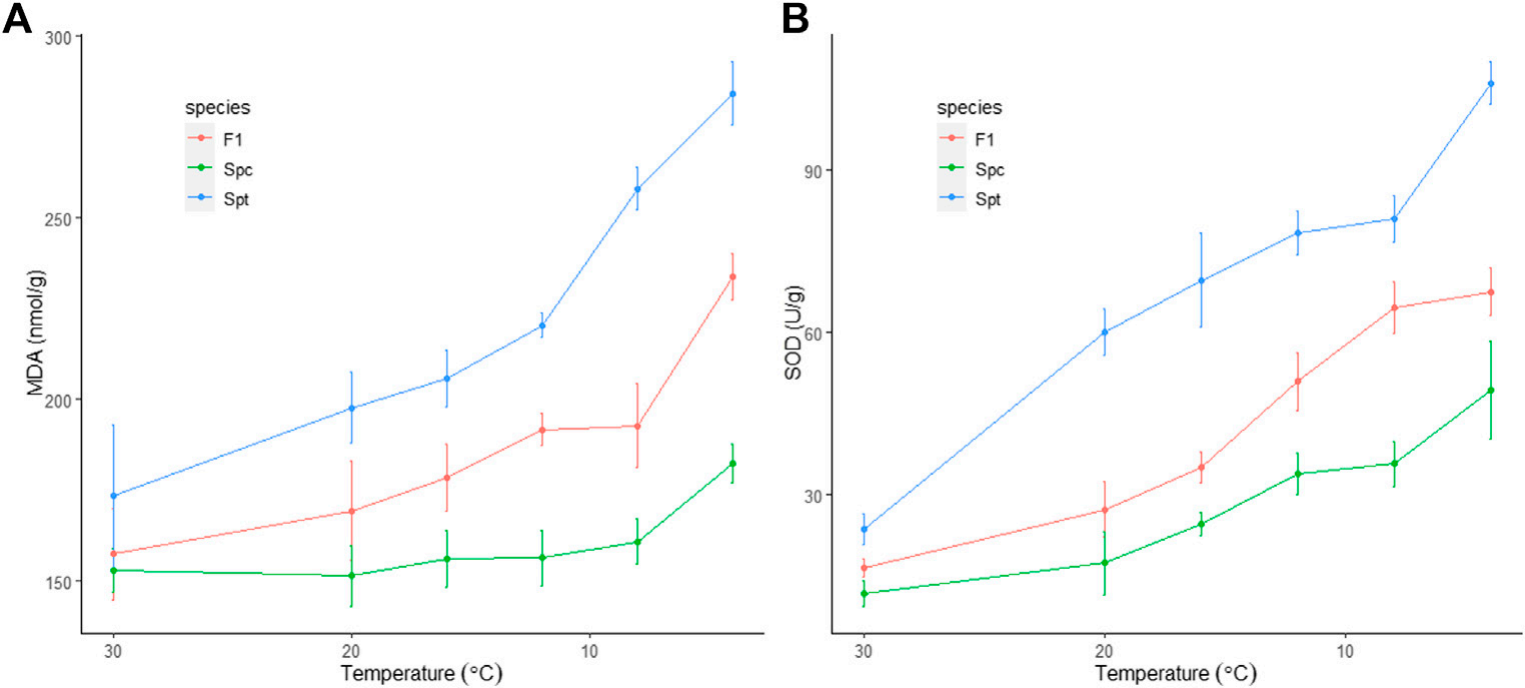
Physiological responses to consecutive chilling stress among the invasive *Sphagneticola trilobata*, the native *S. calendulacea* and their F1 hybrid. **(A)** Malondialdehyde (MDA) concentration; **(B)** Superoxide dismutase (SOD) activity.

During the chilling process, the concentration of malondialdehyde (MDA) increased steadily for both *S. trilobata* (from an average of 186.7 ± 4.0 nmol/g at 30°C to 279.7 ± 6.3 nmol/g at 0°C) and the hybrid (from an average of 165.7 ± 8.4 nmol/g at 30°C to 245.1 ± 2.9 nmol/g at 0°C). In contrast, no significant increase was observed in *S. calendulacea* until 4°C (from an average of 152.6 ± 2.1 nmol/g at 30°C to 190.6 ± 3.6 nmol/g at 0°C) (Figure 1A). As an indicator of membrane injury, the increase in the concentration of MDA is proportional to the degree of cellular metabolism deterioration, indicating that the native *S. calendulacea* suffered the least under cold stress compared to the exotic *S. trilobata* and the hybrid*.* The enzyme superoxide dismutase (SOD) can scavenge the reactive oxygen species (ROS) induced by cold stress and alleviate the damage to membrane, so that the activity of SOD enhances with cold stress. In this study, the SOD activity of *S. trilobata* increased from 23.6 ± 1.1 U/g at 30°C to 106.1 ± 1.6 U/g at 4°C, well above the levels of both *S. calendulacea* (from 11.8 ± 1.0 U/g at 30°C to 49.3 ± 3.7 U/g at 4°C) and the hybrid (from 16.6 ± 2.3 U/g at 30°C to 67.5 ± 1.8 U/g at 4°C) (Figure 1B). The two measurements related to membrane integrity showed the same physiological changes dynamics under consecutive chilling, in which the F1 hybrid showed persistent intermediate chilling tolerance and moderate resemblance to the cold-tolerant *S. calendulacea.* No heterosis with cold tolerance was observed from the physiological properties of the F1 hybrid.

### Transcriptome Assembly and Annotation

After filtering a total of 123.5 million and 151.2 million pooled raw read pairs of one individual under 3 chilling treatments for *S. calendulacea* and *S. trilobata* respectively, 116.2 million and 143.0 million read pairs were fed into Trinity for normalization at a maximum coverage of 50 for the assembly, respectively (Supplementary Table S1). The subsequent assemblies resulted in 276,450 and 228,229 transcripts for *S. calendulacea* and *S. trilobata*, respectively. After removal of redundancy using EvidentialGene, the initial assemblies of *S. calendulacea* and *S. trilobata* were reduced to 88,129 with contig N50 of 1305 bp and 74,593 with contig N50 of 1078 bp, respectively (Table 1). The EvidentialGene-reduced transcriptomes were used as references for subsequent analyses. The overall completeness over the 956 conserved BUSCOs for the two EvidentialGene-reduced assemblies of *S. calendulacea* and *S. trilobata* was 95.0 and 89.8%, respectively (Table 1). The overall mapping rates of clean reads from any samples of either species to their respective assemblies were >80% (Table 1). For the predicted 56,232 proteins in *S. calendulacea* and 50,124 proteins in *S. trilobata*, 40,546 and 36,670 had significant hits in the UniProtKB/Swiss-Prot database. All these lent support for the plausibility for using the available transcriptome assemblies for subsequent analyses.

**TABLE 1:** Summaries of transcriptome assembly for *Sphagneticola trilobata* and *S. calendulacea*.

	*S. calendulacea*	*S. trilobata*
No. contigs (Trinity output)	276450	228229
No. contigs (EvidentialGene output)	88129	74593
Min. contig (bp)	201	201
Max. contig (bp)	48514	48514
Ave. contig (bp)	955.59	833.91
Percent GC (%)	40.39	41.24
No. residues (bp)	84215076	62203502
Contig N50 (bp)	1305	1078
Transcript length assessment		
No. transcripts >90% coverage	6319	5214
No. transcripts >70% coverage	9197	8156
Average mapping rate (%)	83.4	81.3
BUSCO assessment		
No. complete (%)	909 (95.0)	858 (89.8)
No. complete and single-copy (%)	465 (48.6)	608 (63.6)
No. complete and duplicated (%)	444 (46.4)	250 (26.2)
No. fragmented (%)	23 (2.4)	61 (6.4)
No. missing (%)	24 (2.6)	37 (3.8)
Total BUSCO groups	956	956
No. protein predicted	56232	50214
UniProt/Swiss-Prot	40546	36670
No. annotated transcript (% of total)	55458 (61.9)	51095 (68.5)

### Differential Gene Expression Among *S. calendulacea*, *S. trilobata*, and the F1 Hybrid

**FIGURE 2:**
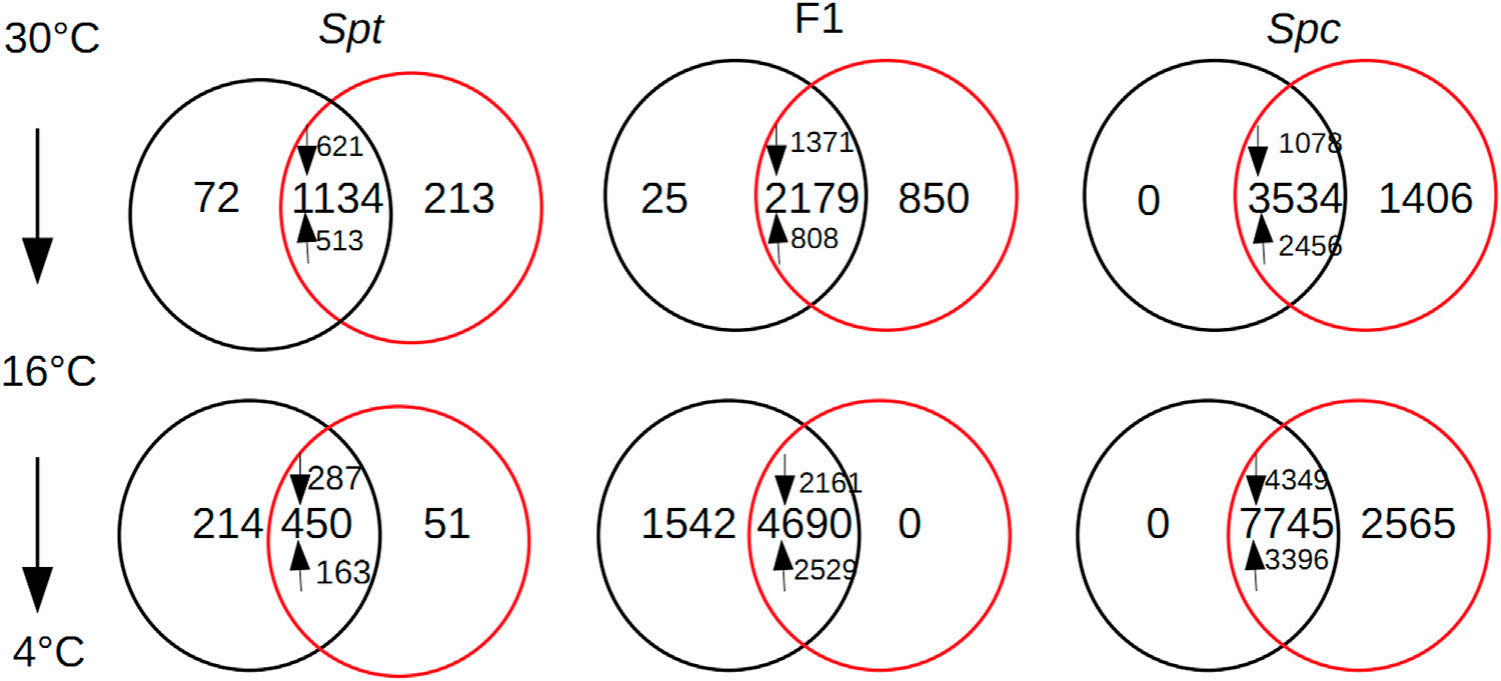
Venn diagram shown for the shared number of differential expression transcripts between the edgeR (red) and DESeq2 (black) for *Sphagneticola trilobata* (*Spt*), the F1 hybrid, and *S. calendulacea* (*Spc*) under three gradient temperature chilling from 30 to 4°C; up-regulated and down-regulated transcripts were marked using upwards arrows and downwards arrows, respectively.

According to the hierarchical clustering based on the Spearman’s rank correlation coefficients, among all the samples under different chilling temperatures, reproducible samples and treatments were validated (Supplementary Figure S1). When individual plants of *S. calendulacea* were transferred from 30 to 16°C, an overlap of 3,534 differential expressed transcripts (DEGs) between edgeR and DESeq2 was identified. Among these DEGs, 1,078 of them were down-regulated, and 2,456 were up-regulated (Figure 2). A total of 646 DEGs (17.1%), consisting of 444 up-regulated and 202 down-regulated transcripts, were identified to be homologous to the 2,637 cold-regulated (COR) genes in Arabidopsis (Park et al., 2015), among which only 42 transcripts were homologous to the 133 genes regulated by three C-repeat binding factors (CBFs) in Arabidopsis (Supplementary Table S2). This tiny fraction of CBF regulons in the COR genes have been validated in previous studies, indicating the indispensable roles for other CBF-independent transcription factors in chilling acclimation (Park et al., 2015; Jia et al., 2016). For the down-regulated transcripts, the photosynthesis (KO00195, Fisher’s exact test, *p*-value: 1.26E-11) and oxidative phosphorylation (KO00190, Fisher’s exact test, *p*-value: 1.80E-4) pathways were significantly enriched (Supplementary Figure S2A). Drastic chilling stress can repress mitochondrial respiration by restricting the activities of oxidative phosphorylation (Kerbler et al., 2018); for warm-climate plants, chilling temperature can lead to substantial reduction in photosynthesis (Allen and Ort, 2001). For up-regulated transcripts, ten KEGG pathways were significantly enriched (Supplementary Figure S2A), with most being associated with secondary metabolites including phenylpropanoids, flavonoids, glucosinolates, as well as primary metabolites including starches, sucrose, and lipid derivatives. These protective compounds or lipid compositions conferred chilling tolerance or cold acclimation *via* plasma membrane stabilization or ROS scavenging (Dempsey et al., 2011; Schulz et al., 2016). One noticeable pathway, circadian rhythm—plant, was also significantly enriched (KO04712, Fisher’s exact test, *p*-value: 4.30E-4); the roles of circadian rhythms in chilling and freezing acclimation have been elucidated for Arabidopsis (Harmer et al., 2000; Fowler et al., 2005). With consecutive chilling from 16 to 4°C, an overlap of 7,745 transcripts identified by two approaches were significantly differentially expressed (Figure 2). Out of these DEGs, 1,030 transcripts homologous to Arabidopsis CORs (369 up-regulated and 661 down-regulated) were identified, of which 78 were homologous to those CORs regulated by CBFs (Supplementary Table S3). For instance, two transcripts *SPC_TRINITY_DN26282_c2_g1_i16* and *SPC_TRINITY_DN26282_c2_g1_i4* homologous to the CBF regulon *AtNUDX2* (At5g47650) were up-regulated significantly at 4°C, which confers enhanced tolerance to oxidative stress in Arabidopsis (Ogawa et al., 2009). Among the 4,349 down-regulated transcripts, two out of the ten enriched KEGG pathways were involved with lipids biosynthesis (biosynthesis of unsaturated fatty acids KO01040, *p*-value: 5.22E-6; fatty acid metabolism, KO01212, *p*-value: 4.06E-2) (Supplementary Figure S2B). This reduction in lipid biosynthesis would induce membrane rigidification, leading to a disturbance of some membrane processes (Li et al., 2015). Pathways related with the removal/repairing of DNA damage under stress were also up-regulated significantly (ribosome biogenesis in eukaryotes, KO03008, *p*-value: 4.43E-5; DNA replication, KO03030, *p*-value: 4.43E-5; nucleotide excision repair, KO03420, *p*-value: 7.88E-3) (Supplementary Figure S2B).

In *S. trilobata*, when temperature dropped to 16°C, fewer transcripts (an overlap of 1,134 transcripts between DESeq2 and edgeR methods) were differentially expressed compared to *S. calendulacea*. However, more functional categories were involved (Figure 2): 150 DEGs homologous to the CORs in Arabidopsis were identified (Supplementary Table S4). The most up-regulated CBF regulon *SPT_TRINITY_DN23330_c0_g1_i3* was homologous to a member of the glutathione S-transferase family (GSTs), EARLY-RESPONSIVE TO DEHYDRATION 9 (*EDR9*) in Arabidopsis (At1G10370). Glutathione S-transferase family proteins play important roles in a wide range of abiotic stresses by alleviating the oxidative damage caused by ROS (Kumar and Trivedi, 2018). For those down-regulated transcripts, the photosynthesis pathway was the most significantly enriched (Supplementary Figure S3A), and reduced photosynthesis is thought to be an indicator of enhanced chilling tolerance (Hannah et al., 2006). With similar functional categories as those in *S. calendulacea*, some secondary and primary metabolism-associated pathways were up-regulated significantly (Supplementary Figure S3A). Since the expression level of genes associated with carbohydrate, amino acid, and secondary metabolism (e.g. flavonoids) were positively correlated with freezing tolerance (Huimin et al., 2015), it indicated that both cold-tolerant *S. calendulacea* and cold-sensitive *S. trilobata* might be well acclimated to moderate chilling. When temperature decreased to 4°C, an overlap of 450 DEGs between two methods were identified (Figure 2), and a total of 76 DEGs (representing 16.9% of the total DEGs, 28 up-regulated and 48 down-regulated) homologous to COR genes in Arabidopsis were identified (Supplementary Table S5). Only one pathway (i.e. carotenoid biosynthesis, KO00906, *p*-value: 3.30E-2) was up-regulated significantly; some carotenoid derivatives have been shown to be bioactive in the acclimation to stress (Havaux, 2014). The lesser up-regulated pathways enriched for S. trilobata compared to *S. calendulacea* (Supplementary Figure S3B) also indicated a lower acclimation capacity associated with attenuated accumulation of cold-responsive metabolites (Cook et al., 2004).

For the F1 hybrid, when temperature decreased to 16°C, a total of 2,179 DEGs (1371 down-regulated and 801 up-regulated) between the two approaches were identified (Figure 2). There were 401 DEGs (18.4% of total DEGs, 202 up-regulated and 199 down-regulated) homologous to the COR genes and 29 DEGs were CBF regulons (Supplementary Table S6). Out of the 401 DEGs, *SPC_TRINITY_DN26922_c2_g4_i1* homologous to *COR27* (At5G42900) was dramatically increased by a log2 fold-change of 13.3; the cold-induction of *COR27* was mediated by the circadian clock (Mikkelsen and Thomashow, 2009). KEGG enrichment analysis for these DEGs revealed that a significant proportion of the up-regulated pathways are involved in primary or secondary metabolites (Supplementary Figure S4A). Plants increase their content of cryo-protective compounds to maximize their cold tolerance (Ramakrishna and Ravishankar, 2011; Arbona et al., 2013). When temperature decreased to 4°C, a total of 4,690 DEGs (2529 up-regulated and 2161 down-regulated) shared by the two analytical approaches were identified (Figure 2). There were 796 DEGs (384 up-regulated and 412 down-regulated) homologous to the COR genes in Arabidopsis, of which 65 were CBF regulons (Supplementary Table S7). In contrast to prior moderate chilling stress, some secondary and primary metabolism-associated pathways were down-regulated significantly, and pathogen response-associated pathways (plant-pathogen interaction, KO04626, *p*-value: 9.52E-7) were the most significantly up-regulated in the F1 hybrid (Supplementary Figure S4B).

### Expression Level Dominance and Homeolog Expression Biases in the F1 Hybrid

**FIGURE 3:**
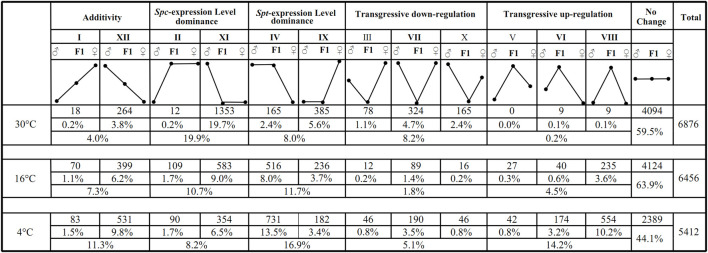
The 13 possible expression patterns of F1 hybrids relative to the paternal parent *S. trilobata* (♂) and maternal parental *S. calendulacea* (♀) upon consecutive chilling stress (30°C, 16°C, 4°C). Each pattern is labelled with the same Roman numeral categorized as in Rapp et al. (2009). Statistically equivalent expressions were indicated with values on the same horizontal line, whereas statistically significant up- and down-regulation were represented by values on higher or lower horizontal lines, respectively. The 13 patterns are binned into five categories representing “no change”, “*Spt*-expression level dominance”, “*Spc*-expression level dominance”, “transgressive expression” and “additivity”, respectively.

Out of the 162,722 transcripts (comprising of 74,593 from *S. trilobata*, hereafter ‘*Spt*’, and 88,129 from *S. calendulacea*, hereafter ‘*Spc*’) that make up the synthetic reference transcriptome for the F1 hybrid, 33,472 *Spt* homeologs and 38,796 *Spc* homeologs were expressed in the hybrid. The reciprocate BLAST resulted in a total of 12,327 pairs of orthologs between the parental species, among which 9,332 homeolog pairs were detected in the F1 hybrid (Supplementary Figure S5). For each homeolog pair, the total expression level in the F1 hybrid was compared to the relative expression level in the two parental species. At 30°C, the expression levels of about 4,094 homeolog pairs (59.5%) in the hybrid were equivalent to those in either parent (Student’s t-test, *p* ≤ 0.05), and were categorized into the class “no change” (Figure 3). The three other expression categories were “additivity” (patterns I and XII, 4.0% in total; Figure 3), “expression level dominance” (patterns II, XI, IV, and Ⅸ, 27.9% in total; Figure 3), and “transgressive expression” (patterns III, VII, X, V, VI, and VIII, 8.4% in total; Figure 3). Only a small fraction of homeolog pairs in the hybrid showed additivity. More homeolog pairs with *Spc*-biased expression level dominance (patterns II and XI, 19.9% in total; Figure 3) were observed compared to those with *Spt-*biased expression level dominance (patterns IV and IX, 8.0% in total; Figure 3). In terms of transgressive expression pattern, more transgressive down-regulation (patterns III, VII, and Ⅹ, 8.2% in total; Figure 3) was observed compared to transgressive up-regulation (patterns V, VI, and VIII, 0.2% in total; Figure 3).

When the temperature decreased to 16 and 4°C, respectively, the proportions of *Spc* homeologs with expression level dominance reduced to 10.7 and 8.2%, respectively. In contrast, the proportions of *Spt* homeologs with expression level dominance increased to 11.7 and 16.9%, respectively (Figure 3). Transgressive up-regulations increased from 0.2% at 30°C to 4.5% at 16°C and 14.2% at 4°C, while transgressive down-regulations decreased from 8.2% at 30°C to 1.8% at 16°C and 5.1% at 4°C (Figure 3). The additivity pattern in the F1 hybrid increased steadily along the chilling process from 4.0% at 30°C to 7.3% at 16°C and 11.3% at 4°C. Overall, a major proportion of homeolog pairs in the F1 hybrid exhibited equivalent expression (from 44.1 to 63.9%; Figure 3), with increases in additivity, transgressive up-regulation, and expression level dominance toward *Spt*, as well as reductions in transgressive down-regulation and *Spc*-biased expression level dominance.

**FIGURE 4:**
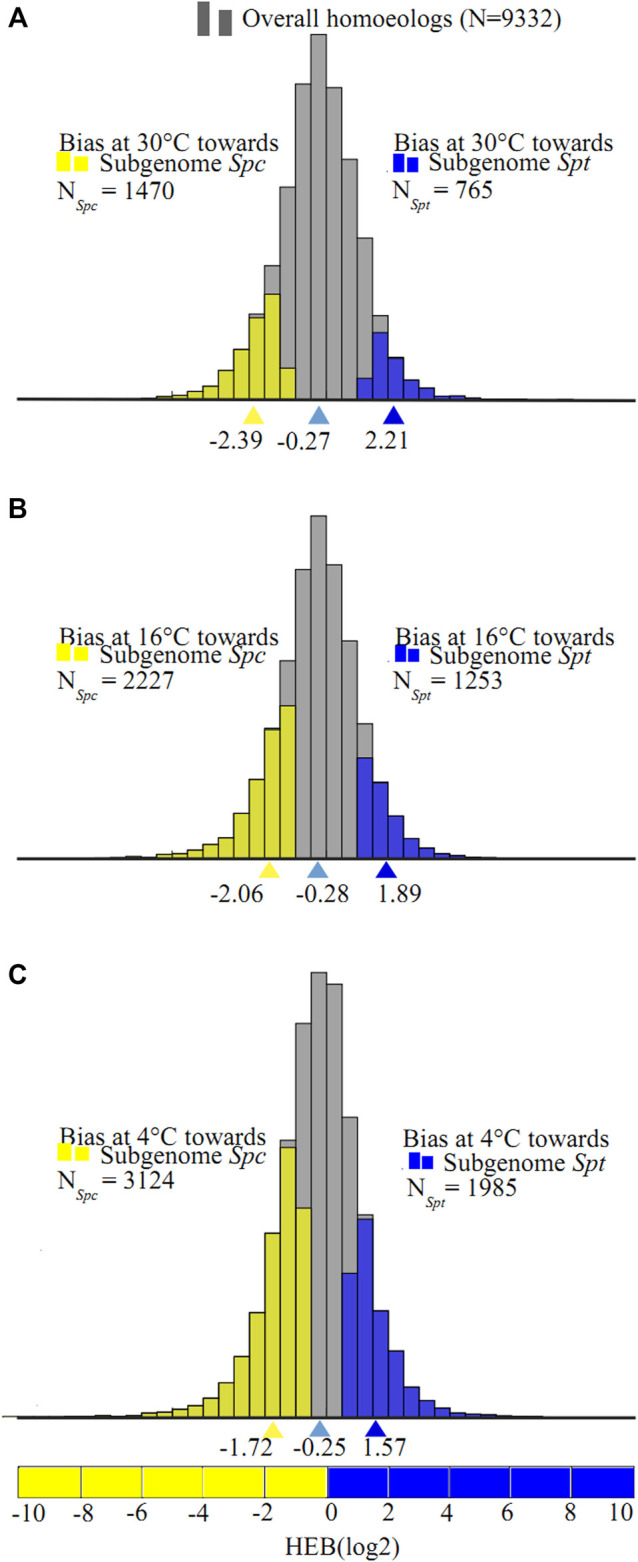
Likelihood ratio test for homeologs expression bias (HEB) in the F1 hybrids between *Sphagneticola trilobata* (*Spt*) and *S. calendulacea* (*Spc*) upon consecutive chilling stress (**(A)** 30°C, **(B)** 16°C, and **(C)** 4°C). The distribution of expression bias (B) for all testable homeologs in the F1 hybrid indicated by gray histograms, and homeolog pairs with significant expression bias toward *Spt-*like homeolog are shown in blue histograms, and those with significant expression bias toward *Spc*-like homeolog are represented by yellow histograms, and the average homeolog bias are marked with corresponding colored triangle values.

Of the 9,332 homeolog pairs in the F1 hybrid, 2,235 pairs showed significant expression bias at 30°C (FDR <0.05 with Benjamini–Hochberg method). Of these biased homeolog pairs, 765 pairs were biased toward *Spt* with $\bar{B}$ = 2.21 (4.6-fold change) and 1,470 pairs biased toward *Spc* with $\bar{B}$ =−2.39 (5.2-fold change) (Figure 4A). At 16°C, more homeolog pairs showed significant bias, with 1,253 and 2,227 pairs biased toward *Spt* ( $\bar{B}$ = 1.89, 3.7-fold change) and toward *Spc* ( $\bar{B}$ = −2.06, 4.2-fold change) respectively (Figure 4B). At 4°C, 1,985 and 3,124 pairs were biased toward *Spt* ( $\bar{B}$ = 1.57, 3.0-fold change) and toward *Spc* ( $\bar{B}$ = −1.72, 3.3-fold change) respectively (Figure 4C). The hybrid showed unbalanced homeolog expression bias with a preference towards the *Spc* subgenome during the chilling process (N_*Spc*_ = 1,470 > N_*Spt*_ = 756, | $\bar{B}$ _*Spc*_ *|* = 2.39 > | $\bar{B}$ _*Spt*_ *|* = 2.21 at 30°C; N_*Spc*_ = 2,227 > N_*Spt*_ = 1,253, | $\bar{B}$ _*Spc*_ *|* = 2.06 > | $\bar{B}$ _*Spt*_ *|* = 1.89 at 16°C; N_*Spc*_ = 3,124 > N_*Spt*_ = 1,985, | $\bar{B}$ _*Spc*_ *|* = 1.72 > | $\bar{B}$ _*Spt*_ *|* = 1.57 at 4°C). Moreover, the increase of biased homeologs from 23.9% at 30°C to 54.7% at 4°C, and the overall average homeolog expression bias values of −0.27, −0.28, −0.25 under the consecutive temperatures, indicated a consistent average bias toward the *Spc* subgenome in the F1 hybrid (Figure 3).

### Likelihood Ratio Test on the Changes of Homeologs Expression Biases in the F1 Hybrid Under Consecutive Chilling

**FIGURE 5:**
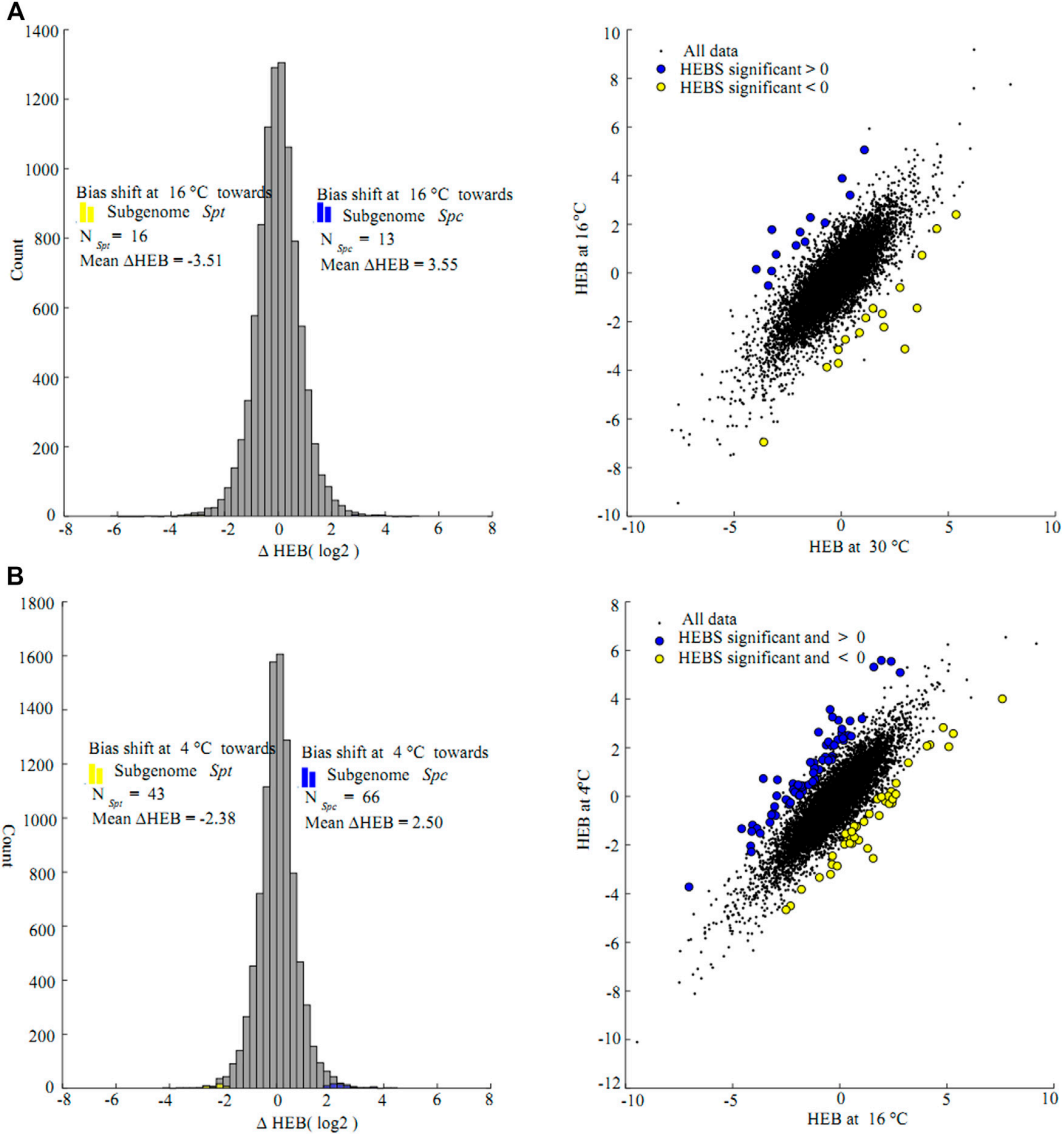
Likelihood ratio test for changes of homeologs expression bias (∆HEB) between consecutive chilling stress (**(A)** 16 *vs*. 30°C **(B)** 4 *vs.* 16°C); in the F1 hybrid between *Sphagneticola trilobata* and *S. calendulacea*. Histogram plot show the distribution of ∆HEB, and the counts of homeolog pairs biased toward *S. trilobata* and *S. calendulacea* marked using blue and yellow histogram respectively; scatter plot of ∆HEB were corresponding to the left histograms. Gene pairs with statistically significant changes in homeolog expression bias marked using the same colors as in the right histograms. Data points in the top-left and bottom-right quadrants represent homeologs where the difference in the bias favors different homeologs at two chilling stress temperatures. The top-right and bottom-left quadrants correspond to homeologs where the difference in bias favors the same homeolog.

Likelihood ratio tests on ∆HEB between consecutive chilling temperatures were conducted on the homeolog pairs in the F1 hybrid. Of the 9,332 pairs of homeologs, only 29 pairs (0.03%) showed significant ∆HEB between 30 and 16°C (Figure 5A). Of these, 13 pairs were biased toward *Spc* homeologs (mean ∆HEB = 3.55, 11.7-fold change) and 16 pairs were biased toward *Spt* homeologs (mean ∆HEB = −3.51, 11.4-fold change) at 16°C compared to at 30°C, and only one pathway enriched for the unigenes biased toward *Spc* homeologs with significant changes in ∆HEB were identified (biosynthesis of secondary metabolites, KO01110, corrected FDR = 0.01, Benjamini and Hochberg method) (Supplementary Tables S8, S9). At 4°C, a total of 109 homeolog pairs (1.2%) showed significant ∆HEB between 4 and 16°C, among which 66 and 43 pairs were more biased towards *Spc* homeologs (mean ∆HEB = 2.50, 5.7-fold change) and *Spt* homeologs (mean ∆HEB = -2.38, 5.2-fold change), respectively (Figure 5B), and no KEGG pathways or GO terms were enriched significantly for these unigenes (Supplementary Tables S10, S11).

In summary, a majority of unigenes in the F1 hybrid were expressed without significant changes in ratio of homeolog expression level under consecutive temperature chilling, displaying an overall balanced pattern of homeolog expression bias. This means that the contribution of homeolog expression bias to cold stress was not clearly evident in the F1 hybrid.

## Discussion

### Extensive Transcriptome Divergence Between Parental *Sphagneticola* Species and Their F1 Hybrid Upon Cold Stress

Cold tolerance is the ability of a plant to tolerate low temperatures without injury or damage. *Sphagneticola calendulacea* and *S. trilobata* were once geographically isolated—the former is native to South China and distributed across temperate and tropical areas, showing considerable tolerance to varying temperatures; in contrast, *S. trilobata* is restricted to tropical areas, and is expected to be cold sensitive. The physiological responses to cold stress between the two parental species and their F1 hybrid demonstrated that while cold tolerance was significantly different between the parental species, transgressive tolerance to cold was not observed in their F1 hybrid, but instead resembled responses of the cold-tolerant *S. calendulacea*.

In concordance with the physiological assays, the transcriptome profiles between the two *Sphagneticola* species and their F1 hybrid revealed that extensive differences in gene expression had occurred. Firstly, contrasting number and functional terms of DEGs were identified upon consecutive cold treatments; the most in terms of numbers and functional terms of induced transcripts were observed in the native *S. calendulacea*, and the least were observed in the invasive *S. trilobata*. Secondly, the overall transcriptome profile of the F1 hybrid resembled the one of the native species *S. calendulacea* upon consecutive cold treatments as evidenced by similar functional categories for those DEGs. Both *Sphagneticola* species and their F1 hybrid seemed tolerant to moderate chilling, but the invasive species *S. trilobata* seemed sensitive to severe chilling as evidenced by relative deficiencies of COR transcripts and associated functional categories.

So far, the CBF-COR signaling pathway was the best characterized regulatory pathway upon cold stress. The CBF/DREB1 transcription factor was found to trigger the expression of downstream COR genes and play crucial roles in the acclimation to cold stress (Shi et al., 2018). However, the CBF regulons made up only a small portion of all the COR genes identified in this study, indicating that regulation of responses to cold is complex, and the role of other CBF-independent pathways should be taken into account (Ding et al., 2019). The expression of CBF transcripts was reported to be rapid and transient, and expression often peaks at 2–3 h after cold treatment (Liu et al., 2017; Ding et al., 2018), whereas the expression of CORs often peaks after 24 h (Thomashow, 1999). As all our transcriptomes were sampled at over 12 h after cold treatment, the CBF transcripts were not found among DEGs in our analysis.

### Expression Level Dominance and Transgressive Expression in the F1 Hybrid During Cold Stress

The null hypothesis for this study was that the total expression levels of the hybrids would equal the average of the parental species. Given significant expression differentiation between parents, if the hybrid was equivalent to one parent (i.e. expression level dominance), the relative contribution of homeologs in the hybrids can be inferred as detailed by Yoo et al. (Yoo et al., 2012). In the chilling processes, the total expression levels of a significant proportion of homeolog pairs (between 44.1 and 63.9%) in the F1 hybrid were equivalent to those of either parent species, and this pattern was consistent with previous studies in cotton (Flagel and Wendel, 2010; Yoo et al., 2012), oilseed rape (Wu et al., 2018), and *Coffea* (Combes et al., 2011; Bird et al., 2018). Similar to previous studies (Bardil et al., 2011; Yoo et al., 2012; Combes et al., 2013; Wu et al., 2018), expression level dominance (see patterns II, XI, IV, and IX in Figure 3) was one of the most prominent forms of expression alterations observed in the F1 hybrids when subjected to cold stress.

Similar investigations on the expression level dominance under different temperatures were reported in *Coffea* allopolyploids (Bardil et al., 2011; Combes et al., 2013). In those studies, a “reversed” expression level dominance under contrasting temperatures was also observed in the studies, but had similar magnitude of expression level dominance (similar proportions of homeolog pairs with expression level dominance). In this study, the proportions of expression level dominance ranged between 22.4 and 27.9%. This might have been caused by different temperature treatments. Instead of the severe chilling simulated in this study, much milder temperatures were used in their studies. Contrary to our expectation, under consecutive chilling, the magnitudes of expression level dominance toward *Spt* in the F1 hybrid increased, whereas those toward *Spc* decreased, although *S. calendulacea* is rather cold-tolerant and *S. trilobata* is cold-sensitive. Yoo et al. (Yoo et al., 2012) proposed an explanation on expression level dominance, for which up- or down-regulation of the homeolog from the non-dominant parent was the most common cause of expression level dominance (the dominant homeolog) in the hybrids or allopolyploids (Yoo et al., 2012). For instance, given the pattern II at 30°C (Figure 3), the expression level of paternal parent *S. trilobata* was lower than the maternal parent *S. calendulacea*. Reconciling with the null hypothesis of additivity, the up-regulation of the *Spt* homeolog in the F1 hybrid gave rise to the expression level dominance in the direction of *Spc* (refer to Figure 3 in (Yoo et al., 2012) for detailed illustration of this explanation). A total of 0.2% unigenes (12 unigenes) exhibited such *Spt* homeolog up-regulation in the F1 hybrid. For the pattern XI at 30°C, the expression level of maternal parent *S. calendulacea* was lower than that of *S. trilobata*, the expression level dominance toward Spc was rendered by the down-regulation of the paternal *Spt* homeolog (non-dominant homeolog). Following this, for the expression level dominance toward *Spt* (patterns IV and IX in Figure 3), steady increasing magnitude of up-regulations (from 2.4% at 30°C, and 8.0% at 16°C to 13.5% at 4°C) and decreasing magnitude of down-regulations (from 5.6% at 30°C, and 3.7% at 16°C to 3.4% at 4°C) of *Spc* homeologs can jointly be attributed (Figure 3). Accordingly, subtle increases of up-regulation (from 0.2% at 30°C, 1.7% at 16°C to 1.7% at 4°C) and sharp decreases of down-regulation (from 19.7% at 30°C, 9.0% at 16°C to 6.5% at 4°C) for *Spt* homeolog diminished the magnitude of expression level dominance toward *Spc* (patterns II and XI in Figure 3).

Generally, under consecutive chilling, the magnitudes of expression level dominance toward *Spt* and *Spc* were reversed, showing increased up-regulation and decreased down-regulation of non-dominant homeologs in the F1 hybrid relative to the pattern of additivity. However, no statistically significant GO terms or KEGG pathways were enriched for unigenes with significant homeolog expression level dominance in the F1 hybrid at 4°C (Supplementary Tables S12, S13). When similar functional enrichment analyses were performed on other categorized unigenes (Supplementary Tables S14–S17), only GO terms in the category of up-regulation were enriched significantly (Supplementary Table S16). Furthermore, most of the enriched GO terms were related to cellular components (e.g. ‘chloroplast’, GO:0009507; ‘plastid’, GO:0009536; ‘chloroplast’, GO:0044434; and so on) (Supplementary Table S16), indicating active recovery of chloroplast content under cold stress for the F1 hybrid. With increased cold stress, the magnitude of transgressive expression was also enhanced significantly (Figure 3). However, the contribution of transgressive up-regulated unigenes to chilling tolerance was not evident phenotypically, as the F1 hybrid exhibited an overall resemblance to the cold-tolerant *S. calendulacea* at 4°C (Figure 1). This may be a result of local adaption; within the sampling location of Guangzhou, the lowest temperatures during winter are often above 4°C and this does not necessarily provide an environment where cold-tolerant alleles are favored by natural selection, even for the supposedly cold-tolerant native species *S. calendulacea.* So far, massive transgressive states were only detected in ancient allopolyploids over an evolutionary timescale, whereby natural selection and cis- and trans-regulatory evolution played a role in their establishment (Rapp et al., 2009; Flagel and Wendel, 2010). This may hint at the relative deficiency of transgressive phenotypes in the F1 hybrid.

### Expression Bias Towards the *S. calendulacea* Homeolog in the F1 Hybrid and Implications for Biological Invasions

With decreasing temperatures, the proportions of expression-biased homeologs increased from 23.9 to 54.7%. These proportions of biased homeologs fell into the range between 15% in synthetic *Brassica napus* allopolyploids (Higgins et al., 2012), and about 70% in *Mimulus* hybrids and allopolyploids (Edger et al., 2018). Teasing apart differences in methodological parameters, organs, and tissues used in different studies, the magnitudes of homeolog expression bias were considered to be associated with some other factors. Firstly, hybridization has greater effects on homeologs expression bias than genome doubling, and transcriptome shock (with manifestations of significant homeolog expression bias and expression level dominance) might occur immediately upon hybridization, but was ameliorated by subsequent genome doubling (Adams, 2007). The relative importance of these two processes to homeolog expression bias have been validated in models such as *Senecio* (Hegarty et al., 2006), *Tragopogon* (Buggs et al., 2011), and *Spartina* (Chelaifa et al., 2010). Secondly, homeolog expression bias was correlated with the extent of subgenome fractionation (unequal gene losses), and significant higher proportions of homeolog expression bias were usually observed in ancient natural polyploids than in synthetic hybrids and allopolyploids (Yoo et al., 2012; Edger et al., 2017; Liang and Schnable, 2018). In this study, newly formed triploid F1 hybrids have emerged since the introduction of *S. trilobata* in South China about 30 years ago, far below the evolutionary timescale for subgenome fractionations, therefore, limited homeolog expression bias was expected in the F1 hybrid. Thirdly, homeolog expression bias in the hybrids or allopolyploids varied with organ type, developmental stage, and type of abiotic stress (Liu and Adams, 2007; Combes et al., 2011; Dong and Adams, 2011), evidenced by the increased proportions of homeolog expression bias in the leaf transcriptomes of the F1 hybrid under consecutive chilling. In summary, the magnitude of homeolog expression bias in the hybrids or allopolyploids seemed to be determined by both intrinsic (e.g. demographic history, organ types, etc.) and extrinsic factors (development stage, abiotic stresses, etc.).

Irrespective of the total expression level in the F1 hybrid, a consistent bias towards the *Spc* homeolog in the F1 hybrid was identified under consecutive cold stress. Such preferential expression for one of the two subgenomes (i.e. unbalanced homeolog expression bias) in hybrids or allopolyploids has been documented in some other allopolyploids including *Arabidopsis suecica* (Chang et al., 2010; Shi et al., 2012), *Brassica* (Wu et al., 2018), *Gossypium* (Yoo et al., 2012), *Tragopogon miscellus* (Tate et al., 2006; Boatwright et al., 2018), and the allopolyploid fern *Polypodium hesperium* (Sigel et al., 2019). Such establishment of subgenome dominance in hybrids or allopolyploids has been ascribed to differences in abundances and distributions of transposable elements (TEs) between progenitors (Freeling et al., 2012; Bird et al., 2018; Edger et al., 2018), and some empirical studies have revealed that the expression level of homeologs were inversely correlated with surrounding density of methylated TEs (Li et al., 2014; Edger et al., 2018). We have roughly estimated the overall proportions of TE in the two *Sphagneticola* species to be 63% in *S. calendulacea* vs. 61% in *S. trilobata* (unpublished data). However, the role of TE differences between *S. calendulacea* and *S. trilobata* on the homeolog expression bias towards the *Spc* homeolog in the F1 hybrid remains unclear. DNA methylation has been found to be sensitive to cold stress in plants (Banerjee et al., 2017), so chilling-induced demethylation might be responsible for the enhanced homeolog expression bias towards the *Spc* homeolog under cold stress. The changes of most homeolog expression bias between consecutive chilling were not significant, and such robust responses to cold stress for the homeolog pairs in the polyploids were also reported in the tetraploid *Arabidopsis kamchatica* (Akama et al., 2014)*,* which were attributed to the shared regulatory network system consisting of diverse transcription factors or epigenetic machineries.

In this study, both the genotypes of the native species *S. calendulacea* and of the resultant F1 hybrids were not under strict natural selection for cold adaptation, given that the sampled population in Guangzhou does not experience extreme cold during winters, and therefore cold-tolerance alleles were not necessarily favored. This led to the observation that the contribution of homeolog expression bias to cold stress was not readily evident in the F1 hybrid. Nevertheless, the northern marginal populations with an extreme winter temperature below zero such as in Shaoguan (13.8°E, 24.8°N), Guangdong Province, should be paid much attention to, where it is expected that cold-tolerance alleles are favored in both *S. calendulacea* and the F1 hybrids, and homeolog expression bias toward the *Spt* homeolog in the F1s is expected to contribute to an enhanced cold tolerance and subsequent invasiveness with range expansion.

## Conclusion

Hybridization is common in plants, and the merging of genomes would mean new combinations of gene regulatory networks and extensive recombination between divergent genomes. Such natural occurrences may give rise to novel phenotypes, leading to ecological diversification and colonization of new niches. Many of the world’s most successful crop species are polyploids and often significantly outperform their diploid relatives. In this study, extensive expression divergences were uncovered between the invasive *S. trilobata* and native *S. calendulacea* upon cold stress, and their F1 hybrid showed a resemblance to the cold-tolerant *S. calendulacea*. Due to the limited sampling, homeolog expression bias to cold stress was not readily evident in the F1 hybrid. Nonetheless, such homeolog expression bias towards the cold-tolerant parental species reflects on the F1 hybrid’s potential robustness towards low temperatures. Overall, given the vigorous growth and the resemblance to the cold-tolerant native species *S. calendulacea*, the F1 hybrid has demonstrated great potential to expand northwards, possibly outperforming the already invasive S. trilobata en route to becoming the next super weed.

## Methods

### Plant Materials, Chilling Treatment, and Physiological Analyses

Cuttings of the native *S. calendulacea* and the invasive *S. trilobata*, as well as their putative F1 hybrid, were collected from a hybrid zone at the coast of Nansha, Guangzhou, China (E113°35′55″, N22°46′44″, alt. 5 m, average monthly temperature ranging between 10 and 33°C, and average day/night temperature ranging between 20 and 28°C). The identities of the samples were morphologically determined in the field and further validated *via* molecular identification using diagnostic genetic markers as described in a previous study (Wu et al., 2013). Voucher specimens of the two parental species and the F1 hybrid were deposited at the Herbarium of Sun Yat-sen University (SYS) with accession numbers ww-20190501–ww-20190503.

Multiple cuttings for all three taxa were allowed to grow into ramets at a common garden in the glasshouse of Zhongkai University of Agriculture and Engineering (Guangzhou, China) for about two and half months, before being transferred into a growth chamber (LRGD-580Y, Hangzhou, China) for subsequent experiments. The chilling treatment simulated a natural gradient cooling, starting at 30°C with 12 h light (150 μE m^−2^s^−1^ illumination from fluorescent lamps)/12 h dark and 60–70% relative humidity. Then, temperature was lowered to 20, 16, 12, 8, 4, and 0°C for a duration of 12 h at each temperature point. Fresh leaves were collected at the end of seven temperature points (30°C as the initial temperature, 20, 16, 12, 8, 4, and 0°C) for physiological analyses, while fresh leaves at three temperature points (30, 16, 4°C) were collected and snap frozen in liquid N_2_ for subsequent RNA experiments.

Chilling injuries experienced by the plants were assayed through measurements of malondialdehyde (MDA) content (using the TBA method) (Raharjo and Sofos, 1993) and superoxide dismutase (SOD) activity (using the WST-1 method) (Peskin and Winterbourn, 2000). Both assays were performed using kits from the Nanjing Jiancheng Bioengineering Institute (Nanjing, China), according to manufacturer’s recommendations.

To avoid wounding effects from consecutive sampling of the same ramet, each ramet derived from one cutting was only used once in each treatment. For both the physiological and RNA experiments, at least three biological replicates were used for each assay.

### RNA-Sequencing, Transcriptome Assembly, and Annotation

Total RNA was extracted from liquid N_2_-frozen leaves using the RNAprep Pure Plant Kit (TIANGEN Biotech Co. Ltd., Beijing, China) and its quality was determined using the Agilent 2100 Bioanalyzer (Agilent Technologies, Palo Alto, CA, United States). Intact RNA was subjected to library construction using the Illumina TruSeq RNA Sample Preparation Kit (Illumina, San Diego, CA, United States) and sequenced on an Illumina HiSeq 2500 to produce 150 bp paired-end reads. The overall quality of the raw sequencing data was evaluated using FastQC ver. 0.11.3 (Babraham Bioinformatics; www.bioinformatics.babraham.ac.uk/projects/fastqc/). Using BBDuk embedded in BBTools ver. 37.76 (jgi.doe.gov/data-and-tools/bbtools), adapter sequences were removed, raw reads with >5% of ‘N’ bases were discarded, bases with Phred Q score <20 were trimmed, and only trimmed reads with lengths of ≥50 bp were retained for subsequent analyses.

The Trinity ver. 2.51 pipeline (Grabherr et al., 2011; Haas et al., 2013) was used for *de novo* transcriptome assembly. The quality-filtered reads generated from different temperature treatments of a single individual were combined and normalized using the Trinity script ‘insilico_read_normalization.pl’ ([max_cov = 50, pairs_together, SS_lib_type = RF]), then used for the *de novo* transcriptome assembly using default parameters. The assembly was performed separately for both parental species, resulting in one species-specific reference transcriptome each for *S. calendulacea* and *S. trilobata*. The tr*2aacds* pipeline of the EvidentialGene package ver. 2017.03.10 (Gilbert, 2013) was used to reduce redundancy of the assembled transcripts, and the “okay” set of transcripts were retained as the final transcriptome assembly. Using TransDecoder ver. 5.30 (https://transdecoder.github.io/), the candidate coding regions within the transcript sequences were identified. Using BUSCO (Benchmarking Universal Single-Copy Orthologs) ver. 3.0.2 (Simão et al., 2015), the completeness of the two *Sphagneticola* reference transcriptomes were assessed against a set of 956 core plant genes. Searching against the protein database UniProtKB/Swiss-Prot using BLASTX [e-value ≤1e-20, max_target_seqs = 1], the counts of full-length (>90% coverage) or nearly full-length (>70%) coding transcripts present in the two reference transcriptomes were summarized using the Trinity script ‘*analyze_blastPlus_topHit_coverage.pl*’. Finally, functional annotation was performed using KOBAS ver. 3.0 (Wu et al., 2006; Xie et al., 2011) to identify gene ontology (GO) and the Kyoto Encyclopedia of Genes and Genomes (KEGG) pathways for these transcripts.

### Differential Expression Analysis Between *S. calendulacea* and *S. trilobata*

Using Kallisto ver. 0.44.0 (Bray et al., 2016), the filtered reads generated from 18 libraries for the two parental species were mapped to their corresponding reference transcriptome assemblies. For the remaining clean reads from 9 libraries of F1 hybrids, a reference consisting of the two parental transcriptome assemblies combined, were used. The transcript abundances were quantified based on transcripts per million (TPM). Only those with TPM (transcripts per million) values of ≥1 for at least one sample were filtered for downstream analyses. Prior to the differential expression analysis, we calculated the pairwise Spearman’s rank correlation coefficients between samples to validate those reproducible samples and treatments. Two R Bioconductor packages, DESeq2 (Love et al., 2014) and edgeR (Robinson et al., 2010), were used to identify the differentially expressed transcripts, and threshold values of 0.001 for false discovery rate (FDR) and 2 for the log2 fold change were used to call up- or down-expressed transcripts. Gene ontology (GO) and KEGG pathway analysis of differentially expressed transcripts was performed using KOBAS ver. 3.0. Following a simplified approach of Krasileva et al. (Krasileva et al., 2013), synthetic assemblies of the two parental species transcriptomes served as reference, and orthologs between parental assemblies represented homeologs to assess homeolog-specific expression (HSE) in the F1 hybrids.

### Reciprocal Best-Hit Orthologs

For the diploid *S. calendulacea* and the tetraploid *S. trilobata*, a 1:2 ortholog relationship was expected when performing reciprocal blast using BLASTX (Altschul et al., 1990) between the two parental species. Stringent criteria of 90% minimum identity and E-values of 1E-50 were used to reduce the number of out-paralogs (i.e. paralogs between species). After those filtering steps, a set of reciprocal best-hit orthologs between the two species were recruited using a custom perl script.

### Expression Level Dominance and Homeolog Expression Bias in the F1 Hybrids

To investigate the relative expression level between the parental species and the F1 hybrid, clean reads from both parental species were mapped to the corresponding reference transcriptomes, while for the hybrids, the combined transcriptomes of two parental species were used as reference. The RPKM (Reads Per Kilobase per Million mapped reads) metric, normalized for sequencing depth and gene length, was calculated for each ortholog pair between species and homeolog pairs in the hybrids. For expression level dominance (ELD) analysis, we compared the total expression level of each homologous pair in the hybrid to that of each gene in the parental species using the Student’s t-test. The differential expression states in the hybrid were categorized into 12 classes according to (Rapp et al., 2009), including additivity, expression level dominance, and transgressive expression. Homeolog expression bias refers to the preferential expression of one progenitor subgenome over the other in the allopolyploid (Grover et al., 2012). According to (Smith et al., 2019), the homeolog expression bias (HEB) in the hybrid can be quantified as:

$$HEB=log\left(M/N\left(\sum_{i=1}^{M}RPKM_{Spt}^{i}/\sum_{j=1}^{N}RPKM_{Spc}^{j}\right)\right)$$

where M and N represent the number of biological replicates for the homeolog pair, while subscripts represent the progenitors of the homeologs. When one uses the base 2 logarithm, HEB = 0 indicates no expression bias between two homeologs in the F1 hybrid, whereas HEB = 3 indicates an 8-fold bias towards homeologs from *S. trilobata*. For any homeolog pair with non-zero read counts in the hybrid, a likelihood ratio test of significant expression bias (the alternative hypothesis, H_1_) against equal expression level (the null hypothesis, H_0_) was implemented for each temperature. In addition, a likelihood ratio test was implemented to determine whether changes in homeolog expression bias was the same (H_0_) or different (H_1_) between consecutive temperature conditions (ΔHEB), following the formula:

$$\Delta HEB=HEB2-HEB1=log\left(\left(\sum_{i=1}^{M}RPKM_{Spt1}^{i}*\sum_{k=1}^{O}RPKM_{Spc2}^{k}\right)/\left(\sum_{j=1}^{N}RPKM_{Spc1}^{j}*\sum_{l=1}^{P}RPKM_{Spt2}^{l}\right)\right)$$;

where M, N, O, and P represent the replicates for the homeolog pair (*Spt* and *Spc*) under temperature condition 1 and condition 2, respectively. The significance of the likelihood ratio test was determined by Chi-square test at the significance level of 0.05. The detailed mathematical descriptions of the likelihood ratio test for HEBs and ΔHEBs following (Smith et al., 2019) and MATLAB codes used in corresponding analysis are available on the Mathworks file exchange (https://www.mathworks.com/matlabcentral/fileexchange/62502).

## Data Availability

The raw reads have been deposited in the NCBI Sequence Read Archive (SRA) under the accession numbers SRR15822809–SRR15822835 under BioProject PRJNA203114. Custom scripts used and transcriptome assemblies and annotations are available at https://github.com/altingia/Sphagneticola_ms_data.
