# Supplementary material for: Expression Level Dominance and Homeolog Expression Bias Upon Cold Stress in the F1 Hybrid Between the Invasive Sphagneticola trilobata and the Native S. calendulacea in South China, and Implications for Its Invasiveness
Source: Front Genet. 2022 May 19;13:833406. doi: 10.3389/fgene.2022.833406 (PMC9160872; doi:10.3389/fgene.2022.833406)
Supplement: Supplementary file 1 [file DataSheet1.PDF]

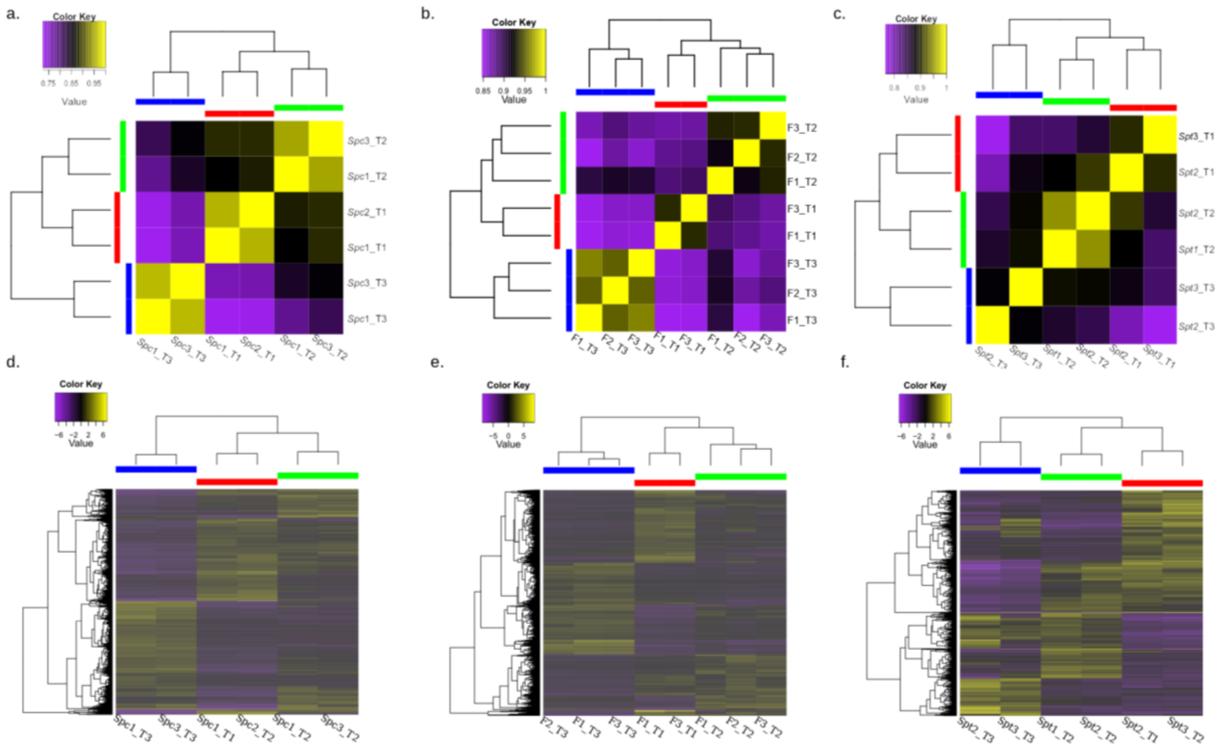

Figure S1. Heatmap of correlation between samples under different chilling stress for *Sphagneticola calendulacea* (a), F1(b), and *S. trilobata* (c) using Pearson's correlation coefficient calculated from the log-transformed raw reads counting per transcripts; Hierarchical clustering dendrogram of expression levels for *S. calendulacea* (d), F1 (e), and *S. trilobata* (f) under different chilling stress. T1, T2, T3 represents 30°C, 16°C, 4°C indicated with red bar, yellow bar, and blue bar, respectively. *S. calendulacea* and *S. trilobata* abbreviated as *Spc*, and *Spt* respectively, and the following numbers mark different biological replicates.

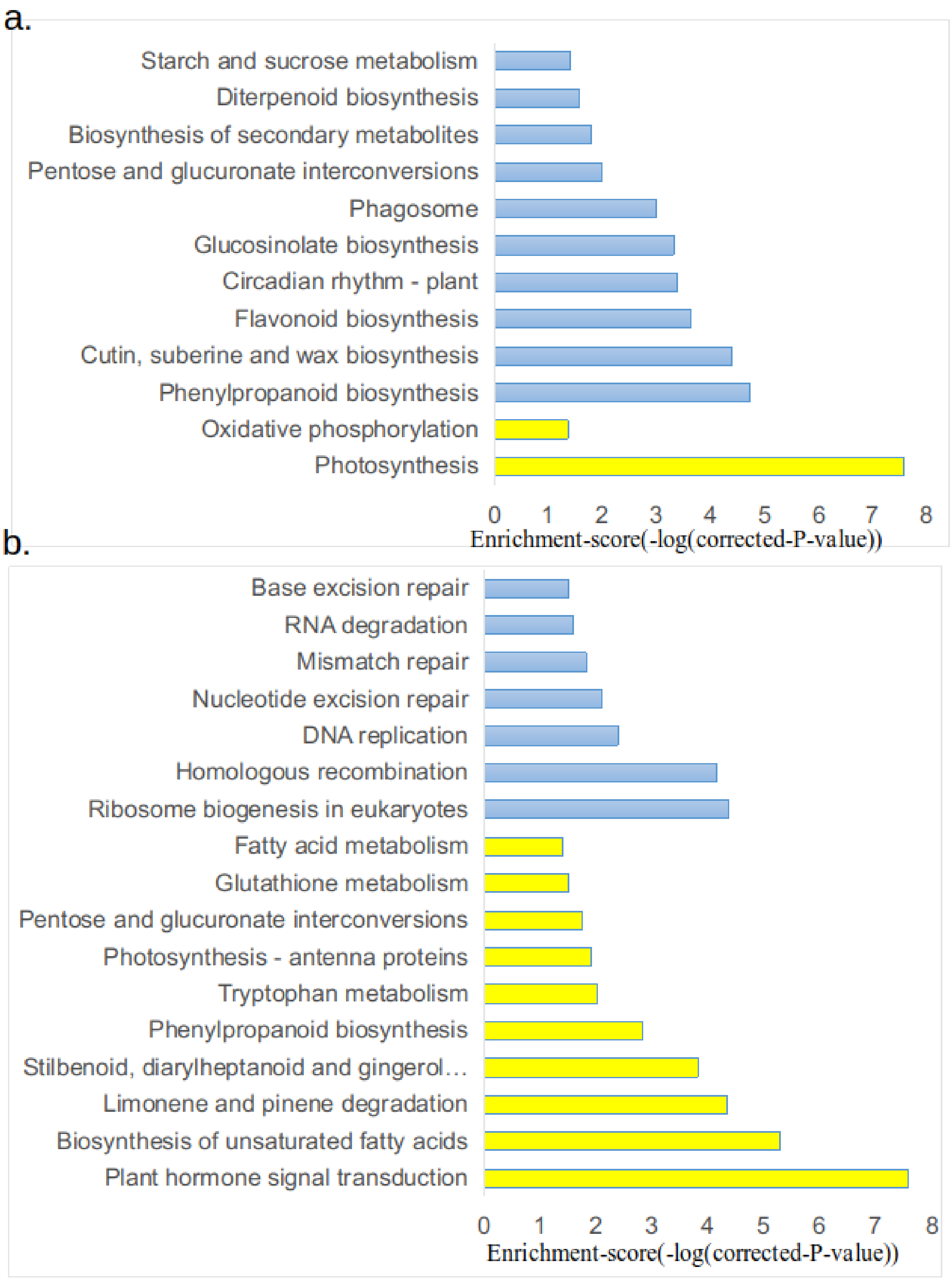

Figure S2. Signficantly enriched KEGG pathways for the differentially expressed transcripts in *Sphagneticola calendulacea* under chilling stress from 30°C to 16°C (a) and 16°C and 4°C (b).The yellow bar and blue bar showed the enrichment scores of down-regulated pathways and up-regulated pathways respectively.

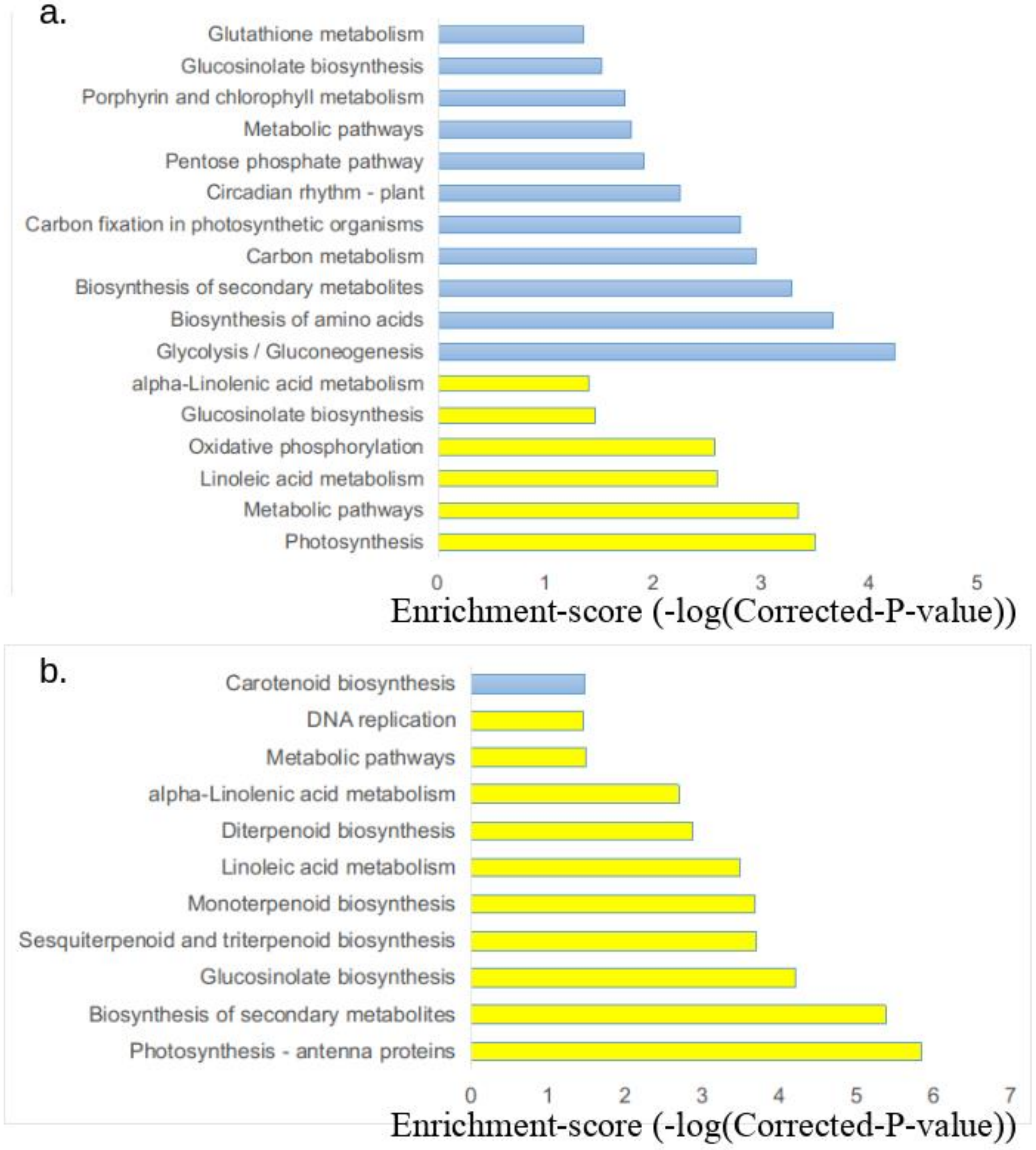

Figure S3. Significantly enriched KEGG pathways for the differentially expressed transcripts in *Sphagneticola trilobata* under chilling stress from 30°C to 16°C (a) and 16°C and 4°C (b).The yellow bar and blue bar showed the enrichment scores of down-regulated pathways and up-regulated pathways respectively.

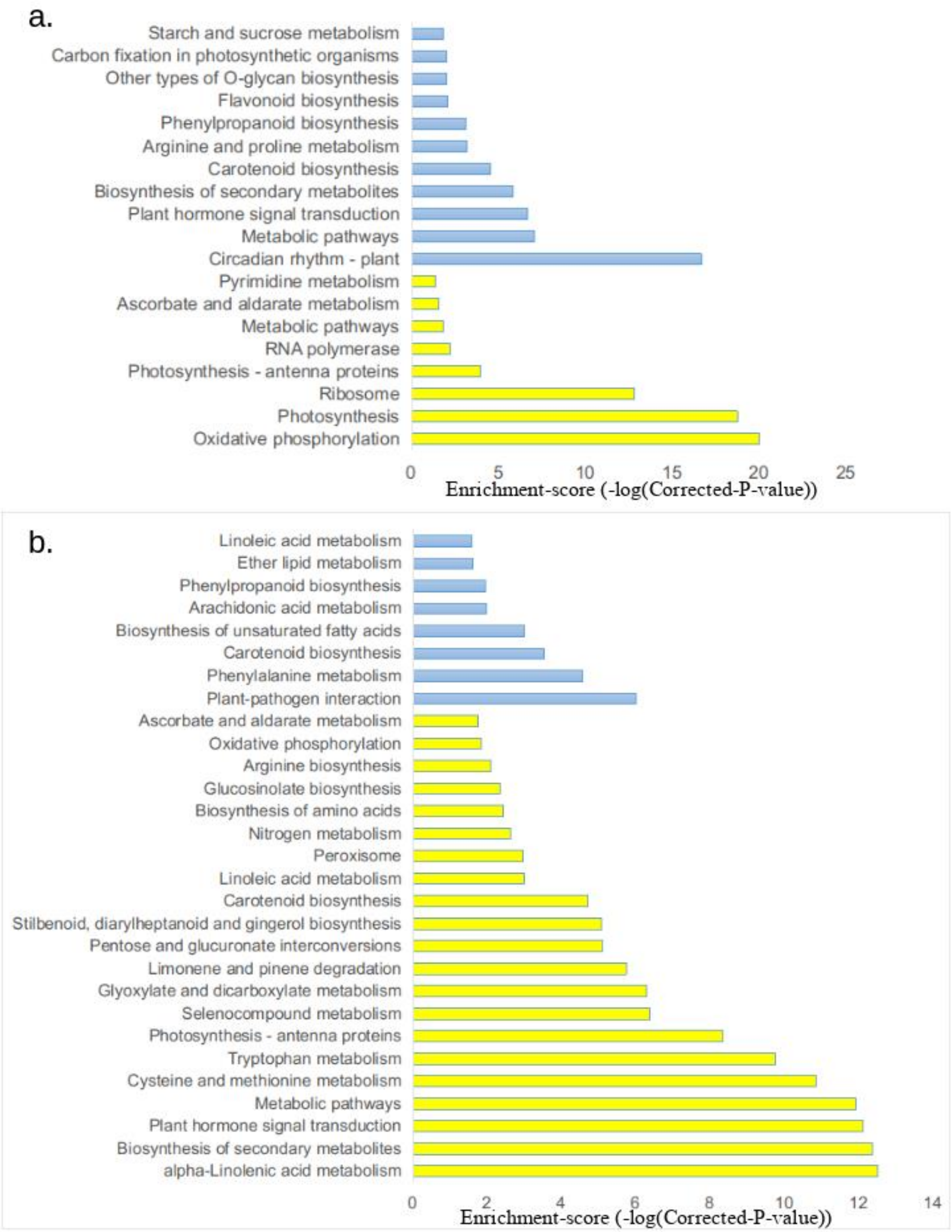

Figure S4. Significantly enriched KEGG pathways for the differentially expressed transcripts in F1s under chilling stress from 30°C to 16°C (a) and 16°C and 4°C (b).The yellow bar and blue bar showed the enrichment scores of down-regulated pathways and up-regulated pathways respectively.

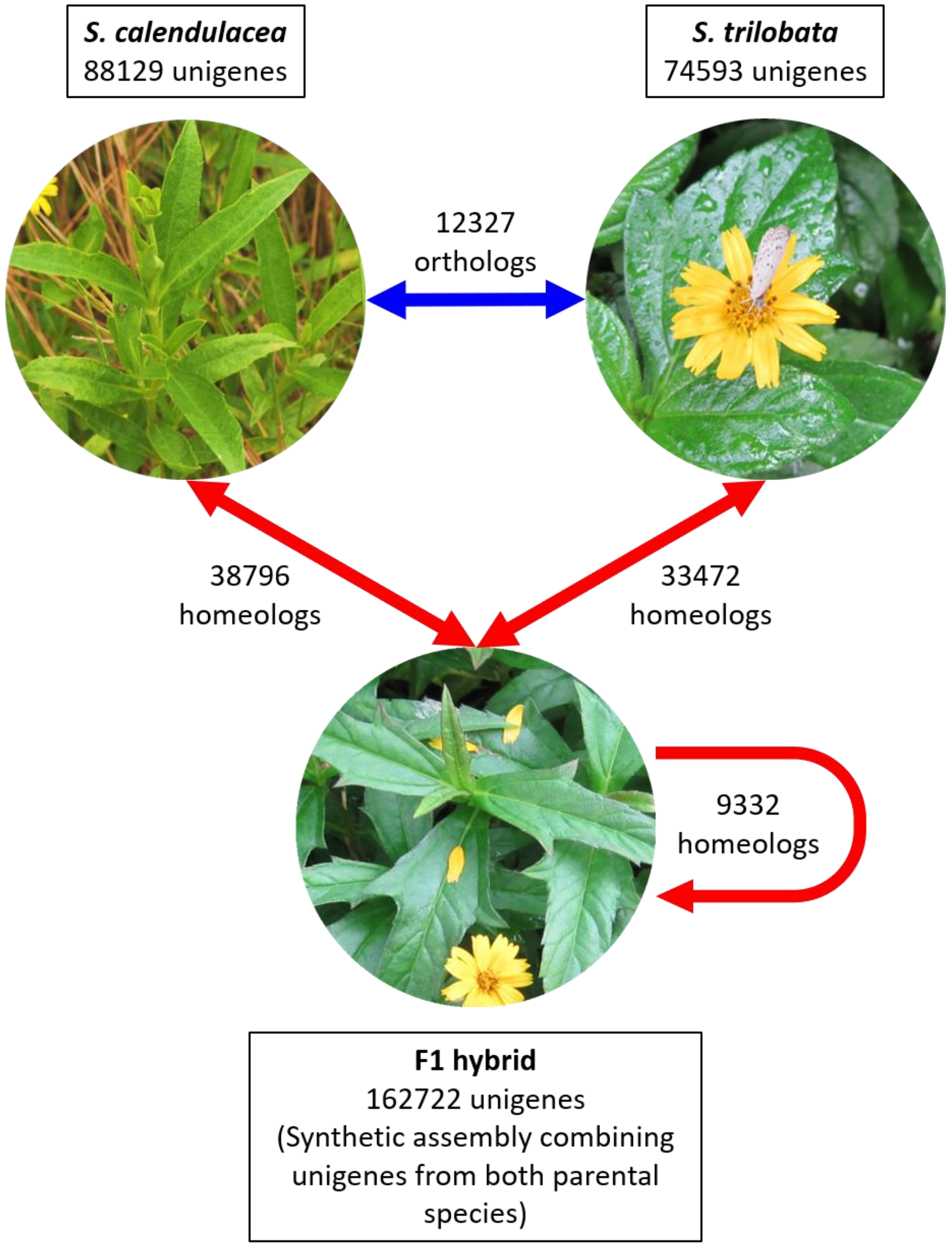

Figure S5. Relationships of orthologs and homeologs among the parental species and the F1 hybrid.
